# ABC Transporters in Cancer Stem Cells: Beyond Chemoresistance

**DOI:** 10.3390/ijms18112362

**Published:** 2017-11-08

**Authors:** Romana-Rea Begicevic, Marco Falasca

**Affiliations:** Metabolic Signalling Group, School of Biomedical Sciences, Curtin Health Innovation Research Institute, Curtin University, Perth WA 6102, Australia; r.begicevic@postgrad.curtin.edu.au

**Keywords:** ABC transporters, cancer stem cells, chemoresistance, cell signalling

## Abstract

The efficacy of chemotherapy is one of the main challenges in cancer treatment and one of the major obstacles to overcome in achieving lasting remission and a definitive cure in patients with cancer is the emergence of cancer resistance. Indeed, drug resistance is ultimately accountable for poor treatment outcomes and tumour relapse. There are various molecular mechanisms involved in multidrug resistance, such as the change in the activity of membrane transporters primarily belonging to the ATP binding cassette (ABC) transporter family. In addition, it has been proposed that this common feature could be attributed to a subpopulation of slow-cycling cancer stem cells (CSCs), endowed with enhanced tumorigenic potential and multidrug resistance. CSCs are characterized by the overexpression of specific surface markers that vary in different cancer cell types. Overexpression of ABC transporters has been reported in several cancers and more predominantly in CSCs. While the major focus on the role played by ABC transporters in cancer is polarized by their involvement in chemoresistance, emerging evidence supports a more active role of these proteins, in which they release specific bioactive molecules in the extracellular milieu. This review will outline our current understanding of the role played by ABC transporters in CSCs, how their expression is regulated and how they support the malignant metabolic phenotype. To summarize, we suggest that the increased expression of ABC transporters in CSCs may have precise functional roles and provide the opportunity to target, particularly these cells, by using specific ABC transporter inhibitors.

## 1. Introduction

Despite progress being made in cancer treatment, patients are still failing therapy, which results in progression of the disease, relapse and an overall reduced patient survival. Although much focus has been placed on the genetic and biochemical mechanisms that cause drug resistance, there is increasing awareness that tumour heterogeneity contributes to therapy failure and eventually disease relapse [1]. Tumours are a complex ecosystem, consisting of a variety of cell types. Heterogeneous intratumoral as well as infiltrating cell types can directly influence tumour cells and create metabolic changes resulting from a hypoxic environment and nutrient fluctuations. These environmental changes, in turn, contribute to the heterogeneous function of malignant cells. Increasing interest in the underlying mechanisms that contribute to tumour heterogeneity is uncovering how these mechanisms are linked to tumour progression, therapy resistance and recurrence. For example, advanced genome sequencing has revealed that cancers are a heterogeneous mixture of genetically distinct subclones that arise through branching evolution [2]. Driver mutations within each subclone can influence cancer cells differently and therefore contribute to functional heterogeneity. In parallel to genetic determinants, strong evidence is emerging that non-genetic determinants, related to developmental pathways and epigenetic modifications, contribute to functional heterogeneity, which is generally ascribed to the maintenance of normal somatic stem cell hierarchies [3]. Epigenetic regulation involves changes in the chromatin structure and function as well as post translational histone modification and DNA methylation. These changes during tumour development are similar to the basic mechanisms governing embryonic development and maintaining adult stem cell hierarchies. The model of hierarchy comes from the concept of normal somatic stem cell tissue regeneration, where cancer stem cells maintain the tumour. Although the hierarchical model for different tumour types is questionable and there is a lot of uncertainty regarding the specificity of the cancer stem cell (CSC) markers used, this model has attracted much interest because of its high clinical relevance. Experimental and clinical studies have indicated that CSCs possess properties that enable them to survive many commonly-employed cancer therapeutics. Indeed, a number of molecular mechanisms can be involved in cancer chemoresistance, such as increased expression of transporters that can extrude anticancer drugs, decreased drug uptake into the cell, activation of detoxification mechanisms, and malfunctioning apoptotic pathways [3,4]. This intrinsic resistance of CSCs to anti-cancer therapy may be the source of disease progression and relapse. Moreover, the properties possessed by cancer stem cells are highly predictive of overall patient survival. Additionally, non-tumour elements within the tumour microenvironment (TME) can influence cancer cells, resulting in significant variations in cellular function [5]. Recent studies point to the potential of the TME to initiate stem cell programs in tumours [6]. Indeed, adaptive drug resistance might be attributable to cross talks between tumour cells and the TME, depending on the context they are in. Collectively, all three mechanisms are strongly linked to therapy failure and tumour recurrence.

Failure of conventional and targeted cancer therapy can occur through an increased efflux of chemotherapeutics, leading to reduced intracellular drug levels and consequently drug insensitivity, usually to multiple agents. A well-established cause of cancer cell multi drug resistance (MDR) is through the increased expression of the ATP binding cassette (ABC) transporter superfamily, which can export a variety of chemotherapeutics out of the cell. However, while the role of ABC transporters in MDR has been established, a less known but emerging theme is the drug efflux-independent role of ABC transporters in cancer biology [7,8]. Increasing awareness has revealed that the loss or inhibition of ABC transporters impacts cellular phenotypes closely linked to differentiation, migration/invasion and malignant potential in a variety of cancers [9,10,11]. Moreover, the loss of ABC transporters in both xenograft and transgenic mouse cancer models can impact tumorigenesis and tumour progression [11,12,13,14]. These contributions are likely related to their normal physiological function of exporting endogenous metabolites as well as signalling molecules [15,16,17]. In this review, we will outline the current understanding of the role played by ABC transporters in CSCs, how they are regulated and how they contribute to tumour maintenance and progression.

## 2. Cancer Stem Cells (CSCs)

Similar to normal tissue, some cancers are organized in an arrangement where tumorigenic CSCs differentiate into progeny, therefore supporting the CSC model [3]. According to this model, tumours are a heterogeneous mixture of genetically distinct subclones that contribute to the functional and phenotypic heterogeneity of the tumour. This results in a hierarchical organization of the tumour cells. At the apex of this hierarchy is a small population of slow-cycling CSCs, endowed with enhanced tumorigenic potential, self-renewal capabilities and an intrinsic resistance to targeted and conventional therapies [18,19,20,21,22,23]. Bayard Clarkson was the first to identify a population of slow-cycling cells which he termed “dormant cells”. These cells were able to escape “anti-proliferative” chemotherapeutics and were assumed to be responsible for leukemic relapse [24,25,26]. The stem cell concept for cancer was first validated by Dick and co-workers who showed that tumorigenic properties can be attributed to a small subset of leukemic cells that are distinguishable from non-tumorigenic cells by the expression of specific surface markers [27]. The first time this concept was applied to solid tumours was in 2003 by Clark and colleagues, when they identified that only a subset of breast CSCs, expressing markers CD44^+^/CD24^−^, are capable of initiating a novel tumour in immunodeficient mice [28]. During the last decade CSCs have been isolated from various solid tumours, including brain, colon, prostate and pancreas [28,29,30,31,32,33] and their phenotypic and functional characteristics have been intensively investigated.

While CSCs and their progeny share the same genotype, they have different epigenetic profiles, resulting in changes to a number of signalling pathways [34,35]. Many of these pathways change to adapt to micro-environmental stresses, including oxygen and nutrient fluctuations, inflammation, low pH and anti-cancer therapies. The challenge in isolating CSCs using markers is to find the right combination of markers. Cell surface markers used to identify CSCs have attracted a lot of controversy due to the high variability between tumours and even within the same tumour [36]. Cell surface markers found in normal stem cells, such as CD133, have been adapted and used [37]. However, CD133 does not characterize CSCs exclusively and whilst it is an indicator, it is not a reliable marker in solid tumours [38]. Other markers of stemness, including Nestin, CD24, CD44, epithelial cell adhesion molecule (EpCAM), Notch 1–4 and Jagged 1–2 have also been investigated. However, epithelial markers can be downregulated, for example, during the process of epithelial to mesenchymal transition (EMT). The EMT process occurs when epithelial cells lose their characteristics and gain a migratory and invasive phenotype to become mesenchymal multipotent stromal cells. This partial EMT phenotype could possess the highest plasticity to adapt to the secondary site conditions. EMT markers include Slug, Snail, TWIST and ZEB1. Adding to the controversy is evidence that terminally differentiated cancer cells can de-differentiate into pluripotent CSCs [39,40]. It is possible that multiple CSC populations co-exist within a tumour and that CSC properties can be attributed to stable and transient cell populations, which can be influenced by environmental factors.

CSCs must be able to self-renew and be pluripotent. Self-renewal markers such as nanog homeobox NANOG, octamer-binding transcription factor 4 (OCT4) and sex determining region y-box 2 (SOX2) are required for the maintenance of stem cell pluripotency. In addition to surface markers, functional CSC markers can be used to isolate CSCs such as increased ABC transporter and aldehyde dehydrogenase (ALDH) activity. Functional identification of CSCs from the bulk tumour is obtained through subpopulations dissociated to single cells and isolated based on a specific combination of markers. These cells are transplanted into immunodeficient mice to determine which cells are capable of initiating a novel tumour [41]. The tumours are identical in histology to the original tumours and have an enhanced tumorigenic potential [30,41]. Furthermore CSCs are quiescent and therefore spend the majority of their time in G0 [20]. Additionally CSCs have an enhanced resistance to conventional and targeted therapies. One striking feature of CSCs is they express high levels of specific ABC transporters. ABC transporter activity can be measured by using fluorescent dyes, such as Hoechst 33342 and rhodamine 123, which can be exported by ATP-binding cassette subfamily-B member 1 (ABCB1) and ATP-binding cassette subfamily-G member 2 (ABCG2) respectively [42]. This flux can be measured by flow cytometry and during analysis this population of cells can be visualized as a negatively stained population of cells, just to the side of the main population, or the side population (SP). Because CSCs efflux these fluorescent dyes they can be sorted by collecting cells that contain only low levels of Hoechst 33342 fluorescence. When isolated, SP cells are capable of initiating a novel tumour in immunocompromised mice in small numbers and give rise to differentiated progeny. However, this isolation method has limitations, because non-CSCs often express ABCB1 and ABCG2 as well. CSCs represent a small population of cells (2–8%) within the bulk tumour; therefore, when isolating CSCs, consideration needs to be given to the right combination of markers.

## 3. ATP Binding Cassette (ABC) Transporter Structure and Location

ABC transporters represent one of the largest families of transmembrane proteins, with 49 members classified into seven gene subfamilies, designated *ABCA–G*. They are found in all living organisms, from microbes to humans, and their conserved structure and function suggests a fundamental role. ABC transporters are defined by their basic structure, consisting of two nucleotide-binding domains (NBD) and two transmembrane domains (TMD). The four domains can be present within one polypeptide chain, comprising a full transporter or within two separate proteins comprising a half transporter. Typically, each TMD consists of six α-spanning helices, totaling 12 α-helices per transporter [43]. The substrate recognition and binding occurs in the transmembrane domains (TMD), which form a pore like structure that opens up to the extracellular space and spans much of the membrane depth [43]. Most ABC transporters function as active transporters that mediate the transport of substrates across the plasma and intracellular membrane, against a concentration gradient. This activity requires energy derived from ATP hydrolysis, coupled with substrate translocation. The transporter remains in a closed transmembrane structure until the substrate is present and the two NBD bind ATP. ATP gets hydrolyzed to ADP and this causes a conformational change in the protein structure, allowing the substrate to be exported out of the cell. Members of the ABC transporter superfamily have a broad spectrum of physiological activity including detoxification, ATP-binding cassette subfamily-B member 1, multidrug resistance protein 1, permeability glycoprotein (ABCB1/MDR1/P-gp), ATP-binding cassette subfamily-C member 1, multidrug resistance-associated protein 1 (ABCC1/MRP1), defense against oxidative stress and xenobiotics (ABCCs and MRPs), lipid metabolism (MDR3, ABCA and ABCG families), and antigen presentation ATP-binding cassette subfamily-B member 2 and 3, antigen peptide transporter 1 and 2 (ABCB2/TAP1 and ABCB3/TAP2) [43].

## 4. Relevance of ABC Transporters in Cancer Cell Biology

The majority of studies on ABC transporters have focused on their ability to transport cytotoxic chemotherapeutics out of the cell, which contribute to cancer chemoresistance. However, due to their ability to transport a wide variety of substrates, their enhanced expression in CSCs, coupled with an increased mitochondrial ATP output, it has been proposed that in addition to exporting drugs out of the cell, ABC proteins transport cell-signalling molecules that contribute to tumorigenesis [7,8,44,45]. In fact, ABC transporters can transport a variety of substrates, such as peptides, inorganic anions, amino acids, polysaccharides, proteins, vitamins and metallic ions [43,46,47,48]. Several members are known to mediate the efflux of cytotoxic chemotherapeutics and are termed multi drug resistant proteins or MDR. The MDR phenomenon occurs when cancer cells exposed to one anti-cancer drug show resistance to various anti-cancer drugs that are structurally and functionally unrelated. The MDR mechanism occurs when membrane pumps are overexpressed and contribute to MDR. The MDR subfamily of transporters are mainly localized in human tissue of the brain, lung, breast, kidneys, liver, ovaries, prostate, placenta and the pancreas [46] (Table 1) and play a critical role in the protection of normal cells. However, some MDR transporters are expressed more highly in CSCs compared to cancer cells and normal healthy tissue [38,49,50,51,52] (Table 1), which makes these transporters attractive targets for pharmacological intervention. For example, ABCG2 is overexpressed in breast CSCs [53], ovarian CSCs overexpress ABCB1 [54] and malignant melanoma initiating cells (MMIC) express high levels of ABCB5 [55]. Indeed, the overexpression of ABC transporters by CSCs has been proposed to maintain stem cell integrity by protecting these cells against naturally occurring xenobiotics. However, the function of ABC transporters beyond their drug-efflux capacity remains largely unexplored. The very presence of ABC transporters in CSCs from a variety of tissues with a large range of putative substrates suggests that, in addition to having a protective effect against naturally occurring xenobiotics, they are involved in processes essential to the character of the cell.

The most well studied members of the MDR family of proteins are ABCB1 (also known as MDR1 or P-glycoprotein, P-gp), ABCC1 (multidrug resistance-associated protein 1, MRP1) and ABCG2 (breast cancer resistance protein, BCRP). These transporters are expressed in the majority of drug resistant tumours.

## 5. ABC Transporter Regulation by Genes and Signalling Pathways

Several genes and signalling pathways are known to regulate ABC transporters (Table 1). ABCC1 and ABCC4 are transcriptionally regulated by *MYCN* [85]. In contrast, ABCC3 is negatively regulated by *MYCN* [85]. Gene amplification of *P53* might regulate the *ABCB1* gene and reporter construct [104]. *ABCB1* transcription is activated by *P63* and *P73* through indirect interaction with the aminoacyl tRNA-peptidyltRNA-decylated tRNA (APE) site [71]. Transcription factor, *OCT4*, can control genes that code for proteins of the ABC superfamily [72]. The aberrant expression of microRNAs can result in the dysregulation of some stem cell genes [105] that lead to an increase in self-renewal and impaired differentiation of stem cells. Transfecting MCF-7/VP-16 breast cancer cells with miR-326 downregulated ABCC1 expression and increased sensitivity to etoposide and doxorubicin [86]. Moreover, high mobility group A (HMGA1) was found to regulate *ABCG2* promoter activity in ovarian CSCs and knockdown reduced proliferative advantage, spheroid forming efficiency and expression of stemness related genes [97].

Signalling pathways that are involved in stem cell renewal and differentiation include signalling cascades, such as epidermal growth factor receptor (EGFR), Hedgehog (Hh) and Wnt β-Catenin. Oncogenic signalling, such as nuclear factor kappa B (NF-κB), Akt, phosphoinositide 3-kinase (PI3K), cyclooxygenase 2 (COX2) and ABC transporters play a role in regulating stem cell renewal, survival, differentiation and chemoresistance [106]. Pharmacological inhibition of receptor tyrosine kinase 2 (ERBB2) with lapatinib, sensitized breast CSCs to doxorubicin, by inhibiting ABCB1 and ABCG2 [69]. Treatment of PC3 cells with cyclopamine, a Smoothened (SMO) signalling inhibitor, downregulated the expression of ABCB1 and ABCG2. Inhibition of Gli1 decreases *ABCB1* and *ABCG2* gene expression in ovarian cancer and enhances ovarian cancer-specific chemotherapeutic response [76]. The PI3K/Akt signalling pathway regulates ABCG2 transporter activity in glioma stem-like-cells lacking phosphatase and tensin homolog (PTEN) [66]. In addition, ABCB1 is regulated by CD133 and DNA dependent protein kinase (DNA-PK) through the PI3K/Akt-NF-κB pathway in multidrug resistant glioblastoma cells [68]. In osteocarcinoma, specific inhibition of the PI3K signalling LY294002 can inhibit ABCB1 and ABCC4, the stem cell cycle and induce apoptosis [91]. Protein kinase C-epsilon directly regulates ABCB1 expression in renal cell carcinoma stem-like-cells [70].

In addition to signalling pathways, molecules such as flavonoids can modulate ABC transporters [42]. Low molecular weight heparin (LMWH) reduced ABCG2 expression in lung cancer stem cells and a combination of LMWH and cisplatin can overcome resistance and induce apoptosis [107]. Several nonsteroidal anti-inflammatory drugs are potent inhibitors of ABCC4 [92]. In breast cancer, hypoxia inducible factor (HIF1α) induction can enrich the breast cancer stem cell population via interleukin 6 (IL6) and IL8 activation of ABCB1 [77].

## 6. Endogenous Role of ABC Transporters in CSCs

While the toxicological and pharmacological roles of ABC transporters have been extensively investigated primarily for their involvement in drug resistance, less focus has been placed on their endogenous physiological roles. Indeed, increasing evidence suggests that the release of xenobiotics is probably not the main physiological function of ABC transporters [92]. Specifically, ABC transporter function in cancer cell biology has been largely overlooked, with all attention polarized to the area of overcoming chemoresistance [7,8,44,45]. We hypothesize that the tumour-promoting function of ABC transporters includes: (a) the release of signalling molecules and hormones, (b) regulation of the cellular redox status, (c) regulation of membrane lipid composition, (d) release of nutrients and metabolites and regulation of cellular metabolism and (e) paracrine regulation of the tumour microenvironment. Obviously, these functions are not mutually exclusive and distinct transporters may have different proposed functions.

## 7. ABC Transporters as Regulators of the Release of Active Biomolecules

**Figure 1:**
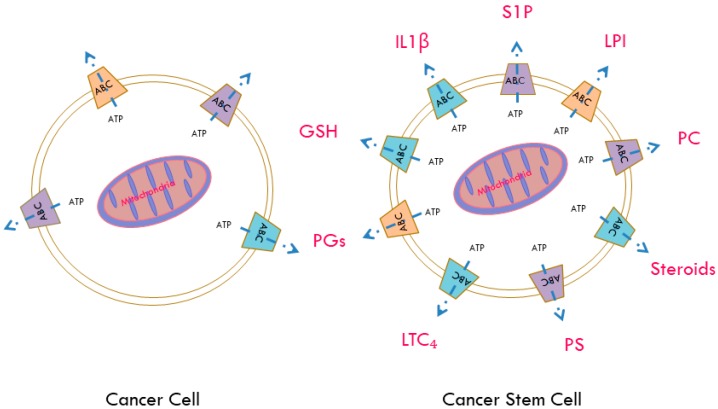
Potential signalling molecules released by ATP binding cassette (ABC) transporters in cancer stem cells. CSCs have an enhanced expression of ABC transporters, coupled with an increased mitochondrial ATP output. ABC transporters primarily use energy derived from ATP to carry out their functions. In addition to exporting a wide variety of drugs, contributing to the multidrug resistant phenotype, ABC transporters can export a variety of signalling molecules that may contribute to an overall enhanced survival advantage. For example, ABCC4 can export prostaglandins (PGs), whereas ABCC1 can export leukotriene C4 (LTC₄), sphingosine-1-phosphate (S1P) and lysophosphatidylinositol (LPI). It was recently found that ABCB5 can export interleukin 1 beta (IL1β) and ABCG2 can export androgens. Members of the ABCA family export phospholipids and have been implicated in intracellular lipid homeostasis. Additionally a variety of ABC transporters can export glutathione (GSH) and conjugates.

ABC transporters have a large variety of putative substrates (Figure 1). For example, ABCC4 has been shown to transport several physiological substrates, including prostaglandins (PGs), cyclic nucleotides, steroid conjugates and folate [92]. Paracrine hormones, such as prostaglandins and structurally related compounds, such as prostacyclin (PGI2), leukotriene’s (LTs) and thromboxane’s (TXs) can be synthesized from phospholipids in a cascade that involves arachidonic acid [108]. Eicosanoids’ primary physiological activity is related to inflammation and they can modulate cardiovascular function, in particular vascular tone. Further cyclooxygenase enzyme, particularly COX2, which is involved in the synthesis of PGs, is overexpressed at sites of inflammation of various human malignancies and in CSCs. Together PGs and LTs can make the vascular endothelium more leaky, suggesting a key role in promoting metastatic potential. Interestingly, ABCC1 can export leukotriene C4 (LTC₄) and ABCC1 deficient mice displayed impaired inflammatory responses attributed to decreased LTC₄ secretion [89]. Furthermore, ABCB5, a marker of skin progenitor cells and malignant melanoma initiating cells (MMIC), functions as a regulator of cellular differentiation [80]. A recent study found that ABCB5 controls interleukin 1β (IL1β) secretion and may serve to maintain MMIC, by pro-inflammatory IL1β/IL8 and CXCR1 signalling [82].

When ATP levels are sufficiently high, ATP–citrate lyase can catalyse the conversion of citrate and coenzyme A (CoA) to acetyl–CoA and oxaloacetate. Acetyl–CoA carboxylase can convert acetyl–CoA to malonyl–CoA, which can go into the fatty acid synthesis pathway. When ATP levels drop, adenosine monophosphate activates AMP-activated protein kinase (AMPK), which regulates fatty acid synthesis by catalysing the phosphorylation of acetyl–CoA carboxylase, thereby controlling the availability of malonyl–CoA. Fatty acids can be used to synthesize signalling molecules, such as phosphoinositides (PIs), eicosanoids and sphingolipids [109]. Work in our laboratory in prostate and ovarian cancer cells has found that the bioactive lysophospholipid lysophosphatidylinositol (LPI) can be transported by ABCC1 [110]. Once released into the extracellular space, LPI activates G protein-coupled receptor 55 in an autocrine manner, initiating a signalling cascade downstream of this receptor that stimulates the proliferation of neighbouring cancer cells [110]. ABCC1 can also transport the bioactive sphingolipid mediator sphingosine-1-phosphate (S1P). Sphingolipids are enriched in lipid rafts in the plasma membrane where they play an important role in signal transduction [90,111,112].

It was recently found that S1P signalling, through the G-protein-coupled-receptor (GPCR) sphingosine-1-phosphate receptor 2 (S1PR2), promotes progenitor survival as well as acinar and endocrine specification in the pancreas [113].

Acetyl–CoA can be converted into acetoacetyl–CoA by A acetyltransferase, which can go into the mevalonate pathway [109]. This pathway is essential in the synthesis of cholesterol esters and steroid hormones, such as androgens, which can be exported by ABCG2. It was recently demonstrated that ABCG2 transporters play an important role in regulating intracellular androgen levels by effluxing androgens in prostate stem cells [101]. Members of the ABCA subfamily have been implicated in critical physiological functions of transmembrane transport of intracellular lipid substrates, such as phospholipids and essential fatty acids. These substrates can be involved in the regulation of differentiation of hematopoietic cells [108]. Looking at the differences in transcript levels of ABC transporters between pancreatic adenocarcinoma and non-neoplastic tissue, Mohelnikova-Duchoneva et al. found that several ABC transporters were upregulated in tumours [57]. Among the upregulated transporters are members of the ABCA family, namely ABCA1 and ABCA7, which implicates a serious impairment of cellular cholesterol homeostasis in pancreatic ductal adenocarcinoma (PDAC) [57]. Shortly after, another study found that the expression of ABCA transporters was associated with poor outcomes in serous ovarian cancer [10]. Using purified and reconstituted ABCA1 and ABCA7 into liposomes for fluorescent-lipid transport studies, Quazi et al. established the substrates for these proteins. They found that ABCA1 actively exported phosphatidylcholine, phosphatidylserine and sphingomyelin, whereas ABCA7 preferentially exported phosphatidylserine [61]. In addition, ABCG1 can also regulate phospholipid transfer [114]. Interestingly, ABCG2 and ABCA7 have been implicated in the transport of amyloid–β peptides in Alzheimer’s disease [63,64,102]. Taken together, the wide variety of drugs, in addition to biologically active substrates, that can be exported by ABC transporters suggests they not only contribute to cancer survival but also to cancer progression. Moreover, the high expression of ABC transporters in CSCs intrinsically links them to tumour initiation, maintenance, progression and metastatic potential. While directly targeting ABC transporters has yielded disappointing results, studying the signalling pathways that contribute to ABC transporter function will be of great relevance for the design of future therapeutic strategies, to overcome resistance and eradicate cancer.

## 8. ABC Transporters and Cellular Redox Status

Oxidative stress occurs when there is a disturbance in the equilibrium between free radicals, reactive oxygen species (ROS) and endogenous antioxidant defence mechanisms, which results in an oxidative environment [115]. ROS can be found in environmental pollutants, smoke and radiation. ROS can also be produced endogenously from mitochondrial metabolism [116]. Oxidative stress can cause damage to the cells’ DNA, proteins and membrane lipids, and elevated ROS have been implicated in cancer initiation and progression [117]. Normal hematopoietic and mammary stem cells maintain ROS at lower levels relative to their mature progeny [118]. This serves to maintain self-renewal and to prevent differentiation. Similarly, CSCs maintain ROS at low levels compared to more differentiated cancer cells, which have higher levels of ROS [118]. This may be attributable to an increase in ROS scavenging molecules that maintain stemness, cancer forming abilities and tumour radio-resistance [118]. Glutathione (GSH) is vital for the maintenance of cellular redox homeostasis and is important for various signalling processes related to proliferation, post-translational modification, immune responses and apoptosis. Cells transport GSH, glutathione disulphide (GSSG) and GSH conjugates (GS-X) in response to oxidative stress and for the purpose of cell detoxification, through membrane proteins. Several ABC transporter members are capable of transporting GSH, including ABCC1, ABCC2, ABCC3, ABCC4, ABCC5, ABCC7 and recently discovered ABCG2 [103]. Mitochondrial transporter, ABCB10, protects cells from increased oxidative stress, which is associated with heme metabolism [119].

Oxidative stress activates NF-κB signalling in CSCs, which initiates transcription of genes involved in proliferation, innate immunity, inflammation and stress responses [120]. Oxidative stress activates nuclear factor (erythroid-derived 2)-like 2 (Nrf2) in the blood brain barrier and the blood spinal cord barrier. This results in increased protein expression and activity of ABCB1, ABCG2 and ABCC2 and involves p53, p38 and NF-κB signalling [121]. Therefore, the CNS barriers are strengthened by oxidative stress which results in reduced drug penetration.

## 9. ABC Transporters and Membrane Lipid Composition

ABCA1 and ABCG1 promote efflux of cholesterol from macrophages to alipoprotein A-1 and high-density lipoproteins and their upregulation preserves macrophage viability, following exposure to oxidized phospholipids and/or apoptotic cells [122]. A few studies have linked lipogenesis to CSCs. Inhibition of fatty acid synthase suppressed breast CSC growth in vivo. ERBB2 positive breast cancer cells were found to be maintained through the peroxisome proliferator-activated receptor (PPARγ) by upregulating de novo lipogenesis [123]. Increased lipid droplets were identified in colorectal CSCs, compared to differentiated cancer cells [124]. Ovarian CSCs contain higher levels of unsaturated lipids [125]. Inhibition of lipid desaturases suppressed sphere-forming ability in vitro and blocked tumour initiating capacity in vivo [125]. Further NF-κB was found to directly regulate the expression of lipid desaturases and inhibiting desaturases blocked the NF-κB pathway [125].

**Figure 2:**
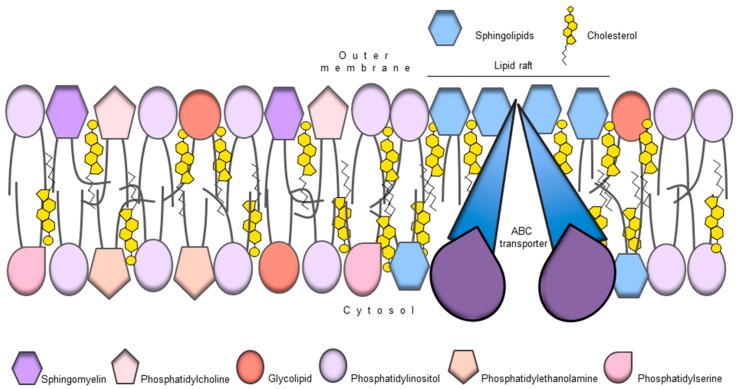
ABC transporters as regulators of membrane lipid composition. ABC transporters can flop lipids from the inner to the outer membrane leaflet, helping to create an asymmetric distribution of lipids between the two membrane leaflets. For example phosphatidylethanolamine and phosphatidylserine are preferentially located in the inner membrane leaflet whereas phosphatidylcholine is preferentially located in the outer membrane leaflet. This distribution may contribute to cell signalling or as structural support contributing to ABC transporter function.

ABC transporters can act as lipid floppases, translocating lipids from the inner membrane leaflet to the outer leaflet (Figure 2). The members of the ABC transporter family—A, B, C, D and G—have been implicated in the transport of lipids or lipid-like molecules, such as steroids, phospholipids and sphingolipids [126]. In addition, the distribution of lipids between the two lipid bilayer leaflets is asymmetric. For example, phosphatidylethanolamine, phosphatidylserine and phosphatidic acid are located in the inner cytosolic membrane leaflet, whereas phosphatidylcholine, sphingomyelin and glycolipids are preferentially situated in the outer membrane leaflet [127]. The preference can stem from physical lipid properties, such as chain length or degree of saturation, as well as head group which can influence membrane architecture and fluidity [128]. While lipids pass through one leaflet to the other by passive diffusion, active transport is required to counteract diffusion-based lipid movement and the resulting homogenization of leaflet content [129]. In addition to being substrates, lipids can act as structural scaffolds for membrane proteins and can be embedded in a protein’s structure [126]. The lipid environment can also influence ATPase or ABC transport activity. Indeed, lipids may act as stabilizers to ABC transporters, allowing for a more efficient coupling between TMD and NBD [126]. Finally, lipids may serve as a reservoir for hydrophobic substrates for multidrug ABC transporters, due to their enrichment in the membrane bilayer [126]. An interesting observation is that drug transport rates were higher in the liquid crystalline phase, compared to the gel phase [130,131]. These findings suggest that the plasma membrane lipid bilayer properties can control the binding and transport of P-gp substrates. The relevance of the membrane phase for P-gp activity is also apparent from the observation that membrane fluidizers and surfactants can reverse MDR [132]. In summary, the importance of lipid metabolism in CSC function has expanded in recent years and the potential direct involvement of ABC transporters is very attractive.

## 10. ABC Transporters and Tumour Metabolism

Metabolic reprograming is an early event in response to a hypoxic environment and elevated anabolic requirements [133]. Cancer metabolism is so important in cancer biology that it has recently become an “emerging hallmark” of cancer [1]. The observation that rapidly dividing cancer cells have defective mitochondria and rely on aerobic glycolysis was first discovered by Warburg et al. [134].

An increase in aerobic glycolysis may optimally serve to support elevated anabolic requirements of rapidly proliferating cells [135,136]. When oxygen supply is abundant, most normal cells metabolise glucose-derived pyruvate to CO_2_ in the mitochondria, a phenomenon termed the Pasteur effect [137]. When the oxygen supply drops, cells switch from mitochondrial to glycolytic metabolism. The master regulator, AMPK, mediates this metabolic switch [138]. In glycolytic metabolism, glucose derived pyruvate is metabolised to lactate. Cancer cells use glycolytic metabolism, even in the presence of oxygen, a phenomenon termed aerobic glycolysis or the Warburg effect, which can nowadays be measured using positron-emission tomography (PET) imaging. As aerobic glycolysis is maintained, oncogenes are activated, notably Kirsten rat sarcoma (*KRAS*) and *MYC*, and tumour suppressor genes, such as *TP53*, are inactivated, which together modulates several biosynthetic pathways to sustain tumour growth [135,139].

The focus of many researchers in the field of cancer metabolism has been on aerobic glycolysis. However, emerging evidence has revealed that tumour cells are a lot more complex than previously thought and aerobic glycolysis represents one aspect of a dynamic metabolic environment in cancers [20,21,140]. Quiescent cancer stem cells seem to prefer mitochondrial metabolism and resemble the metabolism of normal resting cells. Similar to normal cells, quiescent cells use energy produced through mitochondrial oxidative phosphorylation (OXPHOS). Mitochondrial metabolism is not only more efficient at producing ATP (32 molecules) than glycolysis (two molecules) but also generates tricarboxylic acid (TCA) cycle intermediates that are used for macromolecule synthesis. Cancer cells constantly uptake major substrates of metabolism, such as glucose, to increase their ATP output. Indeed, glycolysis can produce ATP more rapidly than OXPHOS with an abundant glucose supply, which in cancer is not always the case.

Although cancers are characterised by poor levels of perfusion, nutrient and oxygen supplies are both temporal and spatial [1]. Blood and oxygen supply is a dynamic process that may leave some areas better perfused than others. In fact, the centre of many solid tumours is very hypoxic and quiescent cells continue to survive with very limited glucose and nutrient supply, limiting the growth of actively proliferating cells. Oxygen concentration is not a limiting factor for mitochondrial respiration until it falls below 1.0 μM [141] and the electron transport chain (ETC) can still function optimally at oxygen levels as low as 0.5% [142]. Therefore, poorly perfused areas of the tumour may not have abundant glucose and nutrient supplies, but have just enough oxygen to allow CSCs to generate mitochondrial ATP and to continue to survive. Hypoxia maintains normal pluripotent embryonic stem cells (ESC) while inhibiting differentiation [143] and creates an environmental niche where stem cells reside. Glioma stem cells, for example, are enriched in hypoxic niches of the tumour [144]. Low oxygen levels can promote both their stemness maintenance and resistance to radio and chemotherapy. Stem properties are maintained through cross talks between transcriptional activity of hypoxia inducible factor (HIF) 1α and 2α and signalling pathways such as Notch, Wnt, and Hh [145,146,147] and increased expression of stemness-related proteins, such as CD133, Nestin and SOX2 [67]. As oxygen levels decrease, proteins related to chemoresistance, such as Ο^6^-methylguanine DNA methyltransferase (MGMT), ABCC1 and ABCB1 increase [67]. While it is known that hypoxia promotes chemoresistance, the mechanisms are not yet fully understood. It has been shown that nucleoside adenosine, which is elevated in the tumour microenvironment, plays a prominent role in mediating resistance mechanisms [148]. Therefore, hypoxic conditions may be a functional component of the stem cell niche, which highlights the close relationship between the maintenance of stem cell phenotype and chemoresistance.

There is also strong evidence to support the idea that mitochondrial function is critical for tumorigenesis [20,149]. This elevated ATP demand may serve to fuel ABC transporters, overexpressed by CSCs, which primarily use energy derived from ATP to transport drugs out of the cell. However, ABC transporters have a wide variety of substrates and energy derived from ATP can be used to transport cell-signalling molecules that maintain the tumour and promote tumorigenesis.

Metabolites produced during mitochondrial respiration, such as citrate, can be acted on by ATP citrate lyase to yield acetyl–CoA and oxaloacetate. If ATP levels are sufficiently high, acetyl–CoA can be used to synthesise fatty acids [109], or to regulate protein acetylation [150]. Currently, the general consensus in cancer metabolism is that cells engage in both glycolytic and mitochondrial metabolism. However, it is important to note that within a tumour, multiple cell populations coexist, a large proportion of which are addicted to glucose [134]. There is also a small population of cancer cells with an impaired TCA cycle or ETC and these cells have a strong reliance on aerobic glycolysis [151,152]. Other cancer cells engage in both pathways robustly and take advantage of their ability to switch from one metabolic phenotype to another to survive treatment, by undergoing therapy-induced senescence [136]. Finally, there are a population of slow cycling CSCs that have a strong reliance on OXPHOS [20,22,140]. Consequently, it would be ideal to target all populations of cancer cells to prevent relapse and eradicate cancer.

## 11. ABC Transporters, Tumour Microenvironment and CSC Niche

The release of bioactive biomolecules can have both autocrine and paracrine targets in the TME. The TME consists of a variety of cell types, including stromal cells, endothelial cells (EC), extracellular matrix (ECM), cancer-associated fibroblasts (CAFs), mesenchymal stem cells (MSCs) and tumour-associated macrophages (TAMs), that can secrete soluble signalling molecules and growth factors. CSCs reside in several anatomically distinct niches, such as the perivascular, hypoxic and the invasive niche. Putative CSC populations in glioblastoma (GBM) and head and neck squamous cell carcinoma (HNSCC) reside in in the perivascular niche, which is located near the invasive tumour edge close to the disorganized and leaky blood vessels [153,154]. The CD133^+^ GBM CSCs produce a high level of vascular endothelial growth factor (VEGF), which induce proliferation of ECs, therefore accelerating angiogenesis. The EC from the blood vessels produce a broad range of cytokines, such as IL6, IL8, EGF and C-X-C motif chemokine 12 (CXCL12), which promote CSC self-renewal, enhance migratory potential and activate pro-survival signalling pathways, such as signal transduction and activation of transcription factor 3 (STAT3), extracellular signal-regulated kinase ERK and PI3K/Akt [155,156].

Due to the compromised vasculature, the inconsistent oxygen supply creates hypoxic areas in solid tumours, such as GBM, which harbours the quiescent CSCs. Hypoxia is one of the key factors that regulates CSC self-renewal, EMT, immune surveillance and resistance to therapy [157]. Activation of HIF in CSCs induces the expression of the metabolic enzyme pyruvate kinase muscle isozyme (PKM2), ABCB1, vascular endothelial growth factor (VEGF) production, angiogenesis, TAM recruitment and CD8^+/−^ T and natural killer (NK) cell suppression [158,159,160]. In addition to oxygen and nutrient deprivation, hypoxic regions of the tumour have high levels of acidification, due to the anabolic switch in tumour cell metabolism and increased lactate production. This environment selects for cells that are able to withstand harsh conditions. Moreover, hypoxia can drive genomic instability by the downregulation of DNA repair pathways, which leads to the accumulation of mutations and acceleration of clonal selection within the CSC pool [161].

Metastasis initiating cells (MIC) are defined by their capability to seed metastatic colonies in secondary organs [162]. The key feature of these cells is their induction of EMT, which can be activated through master transcription factor of the zinc finger E-box-binding homeobox family, including Snail, TWIST and ZEB, by various signals from the tumour stroma, such as platelet derived growth factor (PDGF), ECM and Wnt signalling. High levels of Snail and nuclear β-catenin accumulation were detected at the invasive front of colorectal CSCs [163,164]. Cellular plasticity and the process of EMT is tightly coordinated by epigenetic regulation, including DNA and histone modification, which is highly selective for favourable mutations that drive metastatic tumour traits [165]. These mutations include passenger mutations, such as mammalian target of rapamycin (mTOR) or KDMSC, but also metastasis-specific driver mutations, such as those in the *SETD2* gene [166]. Disseminated tumour cells (DTC) and circulating tumour cells (CTC) seem to prefer homing to the haematopoietic stem cell niche within the bone marrow [167]. Interestingly, these cells can re-enter the circulation to colonise other organs [168]. Infiltrating tumour cells from the primary site secrete cytokine and growth factors to promote the formation of the pre-metastatic niche in the secondary site where MIC seed metastatic colonies [169]. Tumour metastases can be driven by the evolved and selected subpopulations of CSCs. In breast cancer, early metastatic cells possess stem cell-like signatures [170]. Additionally, CSCs and MIC share many common phenotypic and functional properties [171], which provides a strong argument that CSCs are the unit of cancer evolution.

## 12. Conclusions

The targeting of ABC transporters to counteract clinical multidrug resistance in cancer patients has been largely unsuccessful, despite great efforts to develop novel agents. However, the emerging concept that ABC transporters may have a more active role in cancer biology is providing novel impetus to this field of research. Therefore, we propose a shift of focus from the role played by ABC transporters in multidrug resistance to the specific ABC transporter functions in CSCs. One of the limitations is the fact that research has focused on a handful of proteins, namely ABCB1, ABCC1 and ABCG2. Consequently, these transporters are the most investigated in association with CSC properties. However, it would be interesting to investigate the expression profile of all ABC transporters in specific cancer settings to provide a more targeted approach. Particularly attractive, is the therapeutic opportunity to target specific ABC transporters, overexpressed in CSCs, a strategy that will target particularly aggressive and more resistant cancer cells. Another limitation is the lack of knowledge of specific substrates transported and their putative role in cancer biology, particularly in CSCs. Although our understanding of the role played by ABC transporters in CSCs and the specific substrates transported are limited, novel strategies are now available to allow for larger screening. For instance, targeted metabolomics studies have identified novel molecules that may give a specific hint on the role played by these transporters [172,173]. The mechanistic understanding of the endogenous role of ABC transporters in CSC function will be instrumental for assessing their significance in cancer progression and for the development of novel strategies designed to inhibit ABC transporter function in specific cancer settings.

## Figures and Tables

**Table 1:** ABC transporter location, regulation and substrates.

ABC Transporters	Tissue Localization	Expression in Cancer	Expression in Cancer Stem Cells (CSCs)	Regulation by Genes & Signaling Pathways	Exogenous Substrates	Endogenous Substrates
ABCA1	Nervous and hematopoietic system as well as kidney, liver and the blood brain barrier [56]	Pancreas [57], serous ovarian cancer [56]	Serous ovarian cancer initiating cells [58]	Transforming growth factor-β (TGF-β) [59] NF-κB, P65 [60]	Cisplatin [58]	Phosphatidylcholine, phosphatidylserine and sphingomyelin [61]
ABCA7		Pancreas [57]		SREBP2 [62]		Phosphatidylserine [61], amyloid–β peptides [63,64]
ABCB1/MDR1/P-gp	Small intestine, liver, kidney placenta, BBB [56]	Colorectal, liver, renal cancer [56]	Acute myeloid leukemia (AML) [65] glioblastoma [66,67,68] ovaries [54] breast [69] renal cell carcinoma [70]	*P63*, *P73* [71], *OCT4* [72], Mir43b [73], miR-27a [74] hsamiR-451 [75], receptor tyrosine kinase 2 (ERBB2) [69], SMO [76], CD133 and DNA-PK through the PI3K/Akt-NF-κB pathway [68], PKCγ [70], IL6, IL8, hypoxia [67,77]	Anthracyclines actinomycin D, colchicine, etoposide, teniposide, methotrexate, mitomycin C, mitoxantrone, paclitaxel, docetaxel, vincristine, vinblastine [78,79]	Steroids, lipids, bilirubin, bile acids, platelet activating factor [79]
ABCB5	CD133+ progenitor expressed in basal limbal epithelium among epidermal melanocytes [80]	Liver, lung, ovarian, thyroid [56] leukemia cells [81]	Malignant melanoma initiating cells (MMIC) [55,80,82]		Doxorubicin [83], 5-fluorouracil [84], camptothecin [84], irinotecan [84], mitozantrone [84], topotecan [84]	Interlukin 1 beta (IL1β) [82]
ABCC1/MRP1	Lung, testes, peripheral blood monocellular cells [56]	Endometrial, glioma, head and neck, lymphoma, melanoma, renal, thyroid cancer [56]	Glioblastoma [67]	*MYCN* [85], *OCT4* [72], miR-326 [86], hypoxia [67]	Methotrexate, edatrexate, ZD1694, doxorubicin, daunorubicin, epirubicin, idarubicin, etoposide, vincristine, vinblastine, paclitaxel, irinotecan, SN-38, flutamide, hydroxyflutamide [87,88]	Leukotriene C4 (LTC₄) [89], lysophosphatidylinositol (LPI) [44], sphingosine-1-phosphate (S1P) [90], glutathione (GSH), glutathione disulphide (GSSH) [88]
ABCC3/MRP3	Liver, intestine, colon, prostate, testes, brain, kidney [56]	Colorectal, cervical, lung, liver, thyroid, ovarian, pancreatic cancer [56]		*OCT4* [72]	Cisplatin, doxorubicin, etoposide, methotrexate, teniopside, vincristine [88]	GSH [79]
ABCC4/MRP4	Widely-expressed	Prostate, renal, head and neck, endometrial cancer [56]	Osteocarcinoma [91]	*MYCN* [85], *OCT4* [72], PI3K [91]	Topotecan, PMEA, methotrexate, 6-mercaptopurin [88]	Prostaglandins (PGs), cyclic nucleotides, steroid, GSH conjugates and folate [92]
ABCG2/BCRP	Placenta [93], intestine, liver, colon, breast [94]	Cervical, liver, lung, melanoma, testes, breast cancer [56]	Lung [49], pancreas [51,95], liver [96], breast [53,69], ovaries [50,97]	OCT4 [72], miR-212 [98], HMGA1 [97], ERBB2 [69], Hedgehog [99], SMO [76], PI3K/Akt [66]	Mitoxantrone, imatinib, anthracyclins, topotecan, flavopiridol, methotrexate [100]	Androgens [101], amyloid–β peptides [102], GSH [103]

ABC transporters such as ATP-binding cassette subfamily-A member 1 (ABCA1), ATP-binding cassette subfamily-B member 1, multidrug resistant protein 1 (ABCB1), ATP-binding cassette subfamily-C member 1, multidrug resistance-associated protein (ABCC1) and ATP-binding cassette subfamily-G member 2, breast cancer resistance protein (ABCG2) are widely expressed throughout normal healthy tissue. However, some ABC transporters are expressed more highly in cancer cells and some are expressed even more highly in cancer stem cells. A variety of genes and signalling pathways have been implicated in regulating various ABC transporters and they have a variety of exogenous and endogenous substrates.

## Abbreviations

ABC	ATP binding cassettes
CSC	Cancer stem cells
MDR	Multi drug resistance
OCT4	Octamer-binding transcription factor 4
ALDH	Aldehyde dehydrogenase
EpCAM	Epithelial cell adhesion molecule
EMT	Epithelial to mesenchymal transition
SOX2	Sex determining region y-box 2
MMIC	Malignant melanoma initiating cells
P-gp	Permeability glycoprotein
MRP	Multidrug resistance-associated protein
BCRP	Breast cancer resistant protein
APE	Aminoacyl tRNA-peptidyltRNA-decylated tRNA
mTOR	Mammalian target of rapamycin
PAK1	p-21 Activated kinase 1
HMGA	High mobility group A
EGFR	Epidermal growth factor receptor
NF-κB	Nuclear factor kappa beta
PI3K	Phosphoinositide 3-kinase
COX2	Cyclooxygenase 2
ERBB2	Receptor tyrosine kinase 2
SMO	Smoothened
PTEN	Phosphatase and tensin homolog
DNAPK	DNA dependent protein kinase
LMWH	Low molecular weight heparin
HIF	Hypoxia inducible factor
IL	Interleukin
PG	Prostaglandin
PGI2	Prostacyclin
LT	Leukotriene
TX	Thromboxane
LTC₄	Leukotriene C 4
CXCR1	C-X-C chemokine receptor type 1
CoA	Coenzyme A
AMPK	AMP-activated protein kinase
LPI	Lysophosphatidylinositol
PDAC	Pancreatic ductal adenocarcinoma
GPCR	G-protein-coupled receptor
S1P	Sphingosine-1-phosphate
GSH	Glutathione
GSX	Glutathione conjugates
GSSH	Glutathione disulphide
HSC	Hematopoietic stem cells
ROS	Reactive oxygen species
MAPK	Mitogen activated protein kinase
ERK	Extracellular signal-regulated kinase
VEGF	Vascular endothelial growth factor
PPARγ	Peroxisome proliferator-activated receptor
MCT	Monocarbonate transporters
ERK	Extracellular signal-regulated kinase
ESC	Embryonic stem cells
BBB	Blood brain barrier
CNS	Central nervous system
Nrf2	Nuclear factor (erythroid-derived 2)-like 2
HDL	High density lipoproteins
KRAS	Kirsten rat sarcoma
MGMT	Ο^6^-methylguanine-DNA methyltransferase
TCA	Tricarboxylic acid cycle
TIS	Therapy induced senescence
EC	Endothelial cells
ECM	Extracellular matrix
CAF	Cancer associated fibroblasts
MSC	Mesenchymal stem cells
TAM	Tumour associated macrophages
GBM	Glioblastoma multiforme
HNSCC	Head and neck squamous cell carcinoma
EGF	Epidermal growth factor
STAT3	Signal transducer and activator of transcription factor 3
PKM2	Pyruvate kinase M2
NK	Natural killer
MIC	Metastasis initiating cells
SETD	SET domain
DTC	Disseminated tumour cells
CTC	Circulating tumour cells
